# Surgical vs. Conservative Management of Chronic Sciatica (>3 Months) Due to Lumbar Disc Herniation: Systematic Review and Meta-Analysis

**DOI:** 10.7759/cureus.59617

**Published:** 2024-05-04

**Authors:** Ali Hammed, Almonzer Al-Qiami, Hamza Alsalhi, Amjad Almansi, Mahmoud Massoud, Ahmad Alzawahreh, Abdelrahman Hamouda, Christian Tanislav

**Affiliations:** 1 Department of Geriatrics and Neurology, Diakonie Hospital Jung Stilling, Siegen, DEU; 2 Neurological Surgery, Faculty of Medicine, Kassala University, Kassala, SDN; 3 Negida Academy, Medical Research Group of Egypt, Arlington, USA; 4 Faculty of Medicine, Hashemite University, Amman, JOR; 5 Neurology, Prince Hamza Hospital, Amman, JOR; 6 Neurology, Faculty of Medicine, Al-Azhar University, Damietta, EGY; 7 Neurology, Faculty of Medicine, The Hashemite University, Zarqaa, JOR; 8 Department of Neurologic Surgery, Mayo Clinic, Rochester, USA

**Keywords:** conservative management, surgical management, leg vas score, back vas score, lumbar disc herniation, chronic sciatica

## Abstract

Sciatica, characterized by leg or back symptoms along the sciatic nerve pathway, often manifests as a chronic condition lasting over 12 weeks. Decision-making between nonoperative treatment and immediate microdiscectomy for chronic sciatica remains challenging, due to the complex relationship between symptom duration, severity, and lumbar discectomy outcomes. In this systematic review, we conducted a comprehensive search across Scopus, PubMed, Web of Science, and the Cochrane Library, identifying relevant two-arm clinical trials up to September 2023. Rigorous screening and data extraction were performed by two independent reviewers, with study quality evaluated using the risk of bias 2 (RoB) tool. This meta-analysis incorporated four studies comprising 352 participants. Our analysis revealed that conservative treatment was associated with a significant reduction in leg pain and improvement in, SF mental, and physical scores compared to surgical intervention. However surgical treatment demonstrated significant improvement in back pain. In conclusion, our findings suggest that surgical intervention may be more effective than non-surgical treatment for chronic sciatica-related back pain. Conservative treatment significantly reduces leg pain while improving mental and physical health outcomes. Ultimately, our findings support conservative as the initial approach unless surgery is warranted, particularly in cases with neurological deficits or cauda equina syndrome.

## Introduction and background

Sciatica encompasses a wide range of leg and back symptoms, commonly characterized by a sharp or burning pain along the sciatic nerve path extending from the buttock to the leg, and possibly reaching the foot or ankle. [1] Sciatica is a global problem, with lifetime incidence ranging from 13% to 40%. Approximately 90% of instances of sciatica are attributed to lumbar disc herniations (LDH) [2], additionally, it can result from lower back muscle spasms, pyriform syndrome, neural foraminal stenosis, spinal stenosis, and spondylolisthesis [1]. Complete recovery is rare in sciatica caused by LDH, often resulting it prolonged durations of discomfort, and drastically impacting the patients’ quality of life [3]. Albeit rare, if left untreated, it can result in muscle weakness, absence of tendon reflexes or sensory deficits, and bladder dysfunction in some cases.

LDH sciatica treatment options are conservative measures such as physical therapy and pharmacotherapy, or surgical interventions, including discectomy and decompression procedure [4]. Conservative treatment of LDH sciatica can lead to up to a 90% improvement in patients [5], while surgical treatment offers comparable results but is recommended only if symptoms persist following a trial of conservative treatment [6]. However, previous literature failed to show long-term superiority of one approach over the other, even though surgical intervention may offer short-term advantages [7]. Thus, choosing between nonoperative treatment and immediate microdiscectomy, especially for chronic sciatica associated with LDH (>12 weeks) remains a hassle for surgeons and patients [4].

Limited trials comparing surgical to non-surgical treatments for chronic sciatica have hindered the establishment of clear guidelines. Therefore, this systematic review aims to assess the efficacy of surgical intervention versus non-surgical alternatives for individuals with chronic sciatica associated with LDH.

## Review

Methods

Our study followed the widely accepted PRISMA guidelines for systematic reviews and meta-analyses, ensuring the utmost accuracy and reliability of our findings [8]. The meta-analysis was registered on PROSPERO under ID CRD42023469621.

Search strategy and selection criteria

We searched the Cochrane Central Register of Controlled Trials (CENTRAL) (The Cochrane Library Issue 2, 2011), PubMed, SCOPUS, and Web of Science from inception to November 2023 for relevant randomized clinical trials using the following search strategy: (Sciatica OR Sciatic Neuralgia OR Neuralgias, Sciatic) AND (Conservative Treatments OR Treatment, Conservative OR Conservative Management OR Conservative Managements OR Conservative Therapy OR Conservative Therapies OR Epidural Injections OR Steroid Injections OR Nerve Blocks OR Nerve Root Blocks OR Heat Therapy OR Cold Therapy OR Physiotherapy OR Exercise Therapy OR Infrared Therapy) AND (Surgical Interventions OR Operative Procedures OR Operative Procedure OR Surgical Procedures OR Procedure, Surgical OR Surgical Management OR Surgical Managements OR Lumbar Discectomy OR Microdiscectomy OR Laminectomy OR Laminotomy OR Lumbar Decompression).

This study includes only parallel double-arm randomized clinical trials conducted on adult patients (above 18 years) diagnosed with chronic sciatica for more than 3 months due to LDH at the levels of L3-L4, L4-L5, and L5-S1 (Table 1). The comparison was made between patients treated with surgical microdiscectomy and patients with the same diagnosis treated with non-surgical methods such as spinal manipulation, epidural steroid injection, functional education, physiotherapy, or oral analgesics.

**Table 1:** The inclusion and exclusion criteria

Included Criteria for the Study	Excluded Criteria
Parallel double-arm randomized clinical trials, adult patients aged 18 years and above, patients diagnosed with chronic sciatica lasting more than 3 months, patients diagnosed with lumbar disc herniation at the levels of L3-L4, L4-L5, and L5-S1, comparison between patients treated with surgical microdiscectomy and patients treated with non-surgical methods. Non-surgical methods include spinal manipulation, epidural steroidal injection, functional education, physiotherapy, or oral analgesics.	Studies that are not parallel double-arm randomized clinical trials, patients with acute sciatica (duration less than or equal to 3 months), patients with other causes for lumbar pain such as deformity, scoliosis, or any other underlying spinal pathology not related to lumbar disc herniation.

To screen the articles we obtained, we utilized the Rayyan website [9]. Our team of reviewers carefully evaluated the titles and abstracts of the retrieved citations. The article screening process was conducted independently. The eligibility screening process was conducted in two stages: initially, by screening abstracts and subsequently by retrieving and screening full-text articles to ascertain their eligibility for meta-analysis. In the event of any conflicts arising during the screening process, our team resolved them through consensus. Ultimately, we identified four randomized controlled trials (RCTs) that were eligible for data extraction.

The primary outcomes we assessed included the visual analog scale (VAS) for leg pain intensity and back pain intensity. Secondary outcomes included safety outcomes such as adverse events following surgical or non-surgical treatment, and the Short Form-36 (SF-36) mental and physical components were also assessed. For each outcome, data were obtained at 6 weeks (1.5 months), 3 months, and 6 months follow-up durations.

Data extraction and management

Two authors extracted data independently using specially developed data extraction forms. Baseline data were collected on participants, including age, sex, number of participants, number of smokers, level of LDH, duration of pain complaints, and physical status. Primary outcomes (leg pain intensity and back pain intensity), in addition to secondary outcomes (SF-36 mental and physical), were recorded.

Risk of bias assessment

Two authors reviewed the included studies to assess their potential risk of bias (RoB). Rob 2 tool was used. They evaluated each study based on specific criteria such as random sequence generation, allocation concealment, blinding of participants and personnel, incomplete outcome data, selective outcome reporting, and other sources of bias. The authors used the recommended methods by The Cochrane Collaboration to assign a judgment of low risk, high risk, or unclear (due to lack of information or uncertainty over the potential for bias) [10]. Any disagreements between the authors were resolved through consensus, and a third author was consulted if necessary to resolve any remaining disagreements.

Data analysis

We analyzed the study results that are both clinically and statistically homogeneous using the Review Manager software [11]. In this meta-analysis, the Mantel-Haenszel test was used for dichotomous outcomes, and the inverse variance method was used for continuous outcomes in a random-effects model. In cases where there is not enough data for meta-analysis, or if the included studies have diverse outcomes, we presented a narrative synthesis.

Two authors evaluated the study participants, interventions, and outcomes to ensure clinical homogeneity. Meta-analysis was conducted if both authors agreed that the criteria were met. We used the I statistic to measure statistical heterogeneity. When heterogeneity was found, a sensitivity test or meta-regression analysis was warranted.

Results

Study Selection

**Figure 1:**
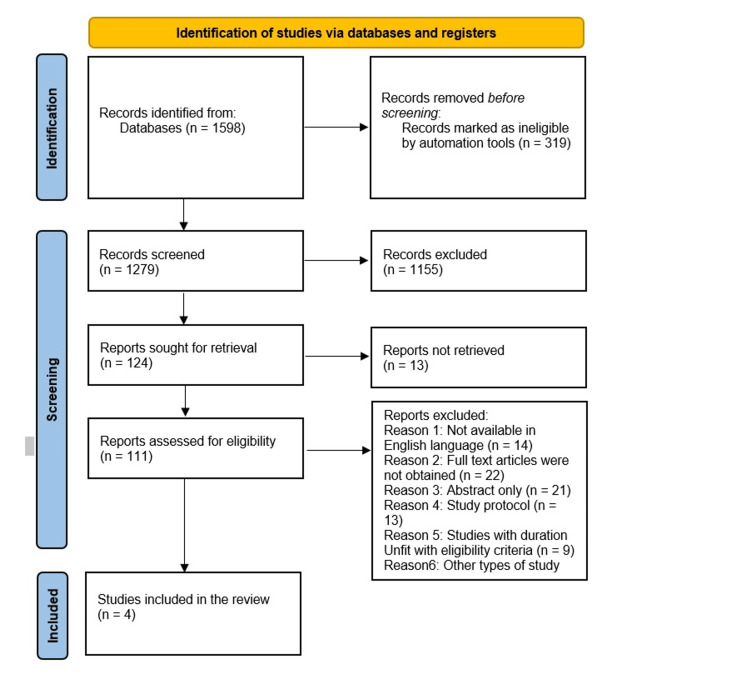
PRISMA flow diagram of the study selection process

As demonstrated in Figure 1, 1598 studies were identified in the initial search, with 1279 remaining after removing duplicates. After screening titles and abstracts, 1155 studies were excluded, resulting in 124 articles for full-text screening. Of these articles, four were included, while 107 were excluded with reasons.

Study and Treatment Characteristics

Study and treatment characteristics are summarized in Table 2. A total of 352 participants (average age 40.4 ± 4.30 years, 202 (57.4) were male) were included in the analysis. The duration of complaints ranged from 3 to 12 months and L5-S1 was the most reported affected spine level. The intervention used across the studies was microdiscectomy, while the comparator involved conservative treatments such as day-to-day functioning education, physiotherapy, oral analgesics, and steroid injections. Additional details are available in Table 2 for a more in-depth examination of the study and treatment characteristics.

**Table 2:** The characteristics of the included studies’ populations

Study ID	Type of Intervention & Comparator		Age (Years) (Mean±SD)		Level of LDH		Gender (Male) N. (%)		Number of Smokers		Medications at Intake		Work Status		Duration of Complaint (Months)	
	Intervention	Comparator	Intervention	Comparator	Intervention	Comparator	Intervention	Comparator	Intervention	Comparator	Intervention	Comparator	Intervention	Comparator	Intervention	Comparator
McMorland et al. 2010 [12]	Surgical microdiscectomy	Epidural steroid/local anesthetic injection	41.9	41.8	L3-L4 (1), L4-L5 (8), L5--S1 (11)	L3-L4 (0), L4- L5 (11), L5--S1 (9)	13 (65%)	11 (55%)	22	23	None (3), Over-the-counter (2), Prescription Nonnarcotic (12), Narcotics (3)	None (3), over-the-counter (1), Prescription Nonnarcotic (13), Narcotics (3)	Employed (8), Medical leave (9)	Employed (8), medical leave (11), unemployed (1)	95±34	55±61
Abd-Elaal et al. 2022 [13]	Microdiscectomy	Day-to-day functioning education, physiotherapy, and oral analgesics, and steroid injections	38.0±8.3	37.1 ± 11.9	L4-L5 (17), L5-S1 (47)	L4-L5 (20), L5-S1 (44)	37 (58%)	39 (61%)	-	-	NSAID: 27, COX-2 Inhibitors: 8	NSAID: 35, COX-2 Inhibitors: 4	Unemployed (related to the disease): 8, unemployed (Unrelated to the disease): 5, employed: 44, disability: 5, student: 1	Unemployed (related to the disease): 8, unemployed (unrelated to the disease): 6, employed: 48, disability: 1, student: 1	7.7 ± 2.9	7.7 ± 2.9
Bailey et al. 2021 [14]	Microdiscectomy	Day-to-day functioning education, physiotherapy, and oral analgesics, and steroid injections	38.0±8.3	37.1±11.9	L4-L5 (17), L5-S1 (47)	L4-L5 (20), L5-S1 (44)	37 (58%)	39 (61%)	-	-	-	-	-	-	7.7 ± 2.9	7.7 ± 2.9
Aronsohn et al. 2010 [15]	Percutaneous microdiscectomy	Epidural steroid/local anesthetic injection	41.4±10.3	51.2 ± 12.4	-	-	16 (63.8%)	10 (44.8%)	8	6	-	-	-	-	3-6 months (3), 6-12 months (5), >12 months (12)	3-6 months (6), 6-12 months (6), >12 months (8)

Risk of Bias

**Figure 2:**
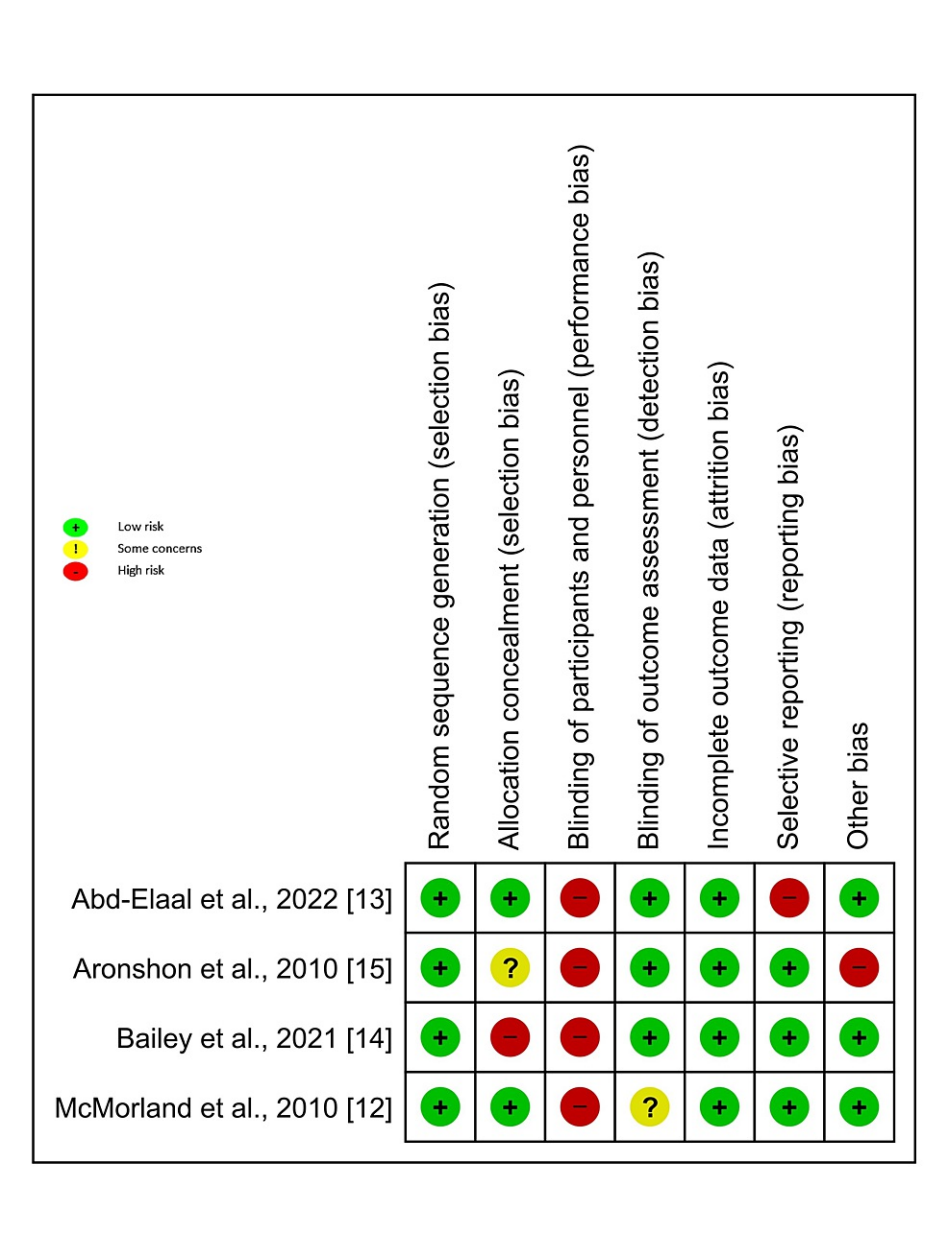
RoB-2 (Quality assessment) RoB: risk of bias RoB summary: Review authors' judgments about each risk of bias item for each included study.

The RoB assessment is demonstrated in Figure 2. The three RCTs [12-14] were of low RoB. One RCT was of moderate RoB [15].

Meta-analysis

Back Pain Analysis

**Figure 3:**
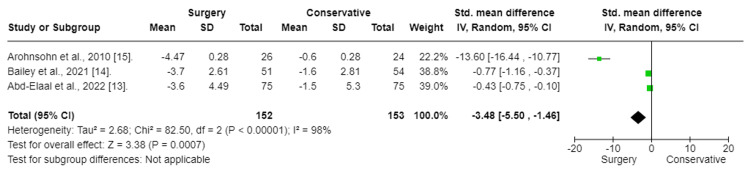
Back pain: Surgery vs. conservative Forest plot of standardized mean differences in back pain symptom VAS score. Std: standard mean difference; SD: standard deviation, CI: confidence interval; df: degrees of freedom; Chi^2^: statistical test for heterogeneity; P: p-value of Chi^2^ (evidence of heterogeneity of intervention effects); I^2^: amount of heterogeneity between trials; Z: test for overall effect; Overall effect P: p-value for significance of overall effect; VAS: visual analog scale.

Three trials reported findings for back pain (Figure 3). Back pain was measured using the VAS score. To assess the effect of the intervention on back pain, a random-mode meta-analysis was conducted using the standardized mean difference (SMD).

**Figure 4:**
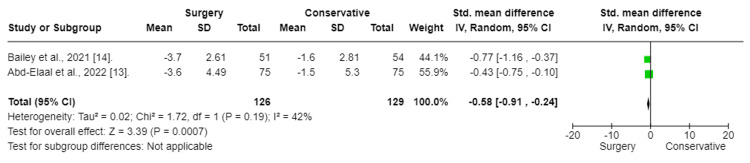
Back pain (Sensitivity test) Forest plot of standardized mean differences in back pain symptom VAS score. Std: standard mean difference, SD: standard deviation, CI: confidence interval; df: degrees of freedom; Chi^2^: statistical test for heterogeneity; P: p-value of Chi^2^ (evidence of heterogeneity of intervention effects); I^2^: the amount of heterogeneity between trials; Z: test for overall effect; Overall effect P: p-value for significance of overall effect; VAS: visual analog scale.

The results of the meta-analysis indicated that surgery had a significant effect and decrease in back pain in patients with chronic sciatica (SMD:-3.82; CI (-5.99 to -1.66); p = 0.0005). To fix heterogeneity, sensitivity tests were conducted, and the Aronsohn study was excluded. The results showed that surgery had a significant effect (SMD: -0.57; CI (-0.92 to -0.22); p = 0.001) (Figure 4). The heterogeneity p-value was 0.17.

Leg Pain Analysis

**Figure 5:**
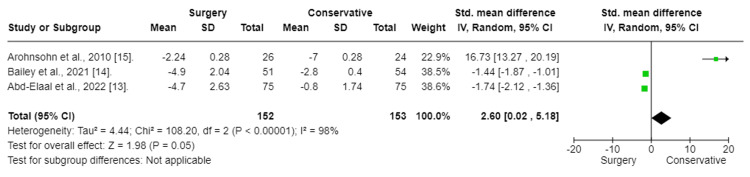
Leg pain: Surgery vs. conservative Forest plot of standardized mean differences in leg pain symptom VAS score. Std: standard mean difference; SD: standard deviation; CI: confidence interval; df: degrees of freedom; Chi^2^: statistical test for heterogeneity; P: p-value of Chi^2^ (evidence of heterogeneity of intervention effects); I^2^: amount of heterogeneity between trials; Z: test for overall effect; overall effect P: p-value for significance of overall effect; VAS: visual analog scale.

**Figure 6:**
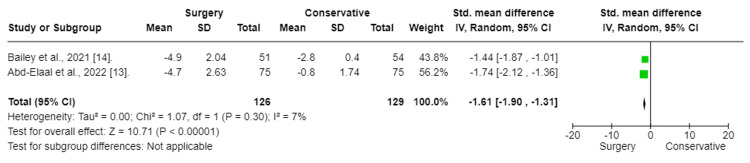
Leg pain (Sensitivity test) Forest plot of standardized mean differences in leg pain symptom VAS score. Std: standard mean difference; SD: standard deviation; CI: confidence interval; df: degrees of freedom; Chi^2^: statistical test for heterogeneity; P: p-value of Chi^2^ (evidence of heterogeneity of intervention effects); I^2^: amount of heterogeneity between trials; Z: test for overall effect; Overall effect P: p-value for significance of overall effect; VAS: visual analog scale.

Leg pain was measured using the VAS score (Figure 5). To assess the effect of the intervention on leg pain, a random-mode meta-analysis was conducted using the SMD. The results of the meta-analysis demonstrated a significant positive impact of conservative treatment on patients experiencing leg pain with chronic sciatica (SMD 2.60; CI (0.02 to 5.18); p = 0.05). In an attempt to resolve heterogeneity, sensitivity tests were conducted, and the Aronsohn study was excluded (Figure 6). The results showed that surgery had a significant effect on leg pain (SMD difference: -1.61; CI (-1.92 to -1.31); p = 0.00001). The heterogeneity p-value was 0.3.

Short Form (SF)-36 Score Mental Analysis

**Figure 7:**
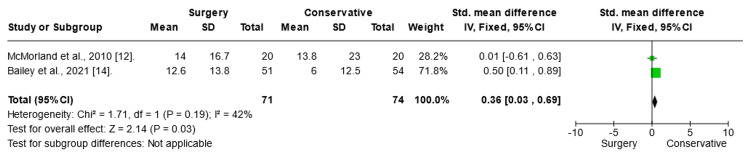
SF-36 mental analysis Forest plot of standardized mean differences in SF-36 mental analysis. Std: standard mean difference; SD: standard deviation; CI: confidence interval; df: degrees of freedom; Chi^2^: statistical test for heterogeneity; P: p-value of Chi^2^ (evidence of heterogeneity of intervention effects); I^2^: amount of heterogeneity between trials; Z: test for overall effect; Overall effect P: p-value for significance of overall effect; SF: short form.

To assess the effect of the intervention on SF-36 scores in mental analysis, a fixed-mode meta-analysis was conducted using the SMD (Figure 7). The results of the meta-analysis highlighted a significant positive impact of conservative treatment on SF-36 mental score in patients with chronic sciatica (SMD= 5.7; CI (1.02:10.37); p = 0.02).

SF-36 Score Physical Analysis

To assess the effect of the intervention on SF-36 scores in physical analysis, a fixed-mode meta-analysis was conducted using the SMD. The results of the meta-analysis revealed a significant improvement in the conservative treatment of SF-36 mental analysis in patients with chronic sciatica (SMD 0.96; CI (0.61:1.30); p = 0.0001).

**Figure 8:**
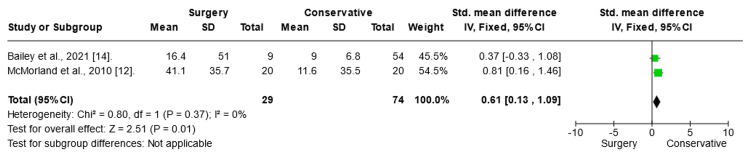
SF-36 score physical analysis Forest plot of standardized mean differences in SF-36 physical analysis. Std: standard mean difference; SD: standard deviation; CI: confidence interval; df: degrees of freedom; Chi^2^: statistical test for heterogeneity; P: p-value of Chi^2^ (evidence of heterogeneity of intervention effects); I^2^: amount of heterogeneity between trials; Z: test for overall effect; Overall effect P: p-value for significance of overall effect; SF: short-form.

Meta-Regression Analysis

To determine the impact of various variables on low back pain including age, BMI, and sex, meta-regression was performed. Analysis showed no association between age (OR (95% CI) = -0.164 (CI (-0.577-0.249), P =0.443), BMI (OR (95% CI) =-9.646 (CI (-0.117-0.08), P=0.322) and sex (OR (95% CI)=-0.214 (CI (-0.544-0.217) P=0.09) and the effect size.

Discussion

Overall Effect of Surgical vs. Non-Surgical Treatment

In the meta-analysis of our included studies [12-15] involving patients with persistent sciatica caused by lumbar disk herniation, conservative treatment may be more effective than surgical intervention in reduction of the leg pain, with a marginal statistical significance (p = 0.05). Back pain analysis suggests that conservative treatment is associated with a remarkable improvement in back pain. Additionally, conservative treatment has a more substantial impact on the SF-36 mental score (SMD = 5.70) and SF-36 physical score, suggesting that it offers long-term benefits for both physical and mental health when compared to surgical intervention. These results underscore the potential advantages of prioritizing conservative approaches over surgery in the management of chronic sciatica stemming from lumbar disk herniation.

Update on Evidence and Significance of the Study

Microdiscectomy-related randomized trials are effective in the management of both leg and back pain. However, this finding is not consistent with our results for leg pain. This discrepancy can potentially be explained by the fact that patients included in our study were required to have symptoms for a minimum of three months [16,17].

Comparing our results with earlier studies, we found that a recently published systematic review revealed that discectomy was initially beneficial, but the effect declined over time compared with either non-surgical care or epidural steroid injections. Generally, discectomy resulted in faster relief of pain and disability, but it was inconsistent over time as it lasted only for 12 months. However, it's important to note that they included in their analysis a wide range of pain-compliant durations, with a majority of studies having durations of less than 3 months, which was the minimum eligible duration for inclusion in our study [18].

The evaluation of the quality-of-life outcomes post-intervention is of utmost importance in guiding therapeutic decisions. Our study has revealed surprising and meaningful results, indicating that conservative therapy is associated with superior improvement regarding quality of life compared to surgical modalities. This finding emphasizes the importance of considering not only clinical outcomes but also the impact on patients' overall well-being. The observed benefits in quality of life after conservative therapy suggest that it may be a preferable and patient-centered approach in certain cases. These results highlight the need for a comprehensive assessment that goes beyond traditional clinical measures, recognizing the holistic impact of interventions on individuals' lives.

Surgical treatment for sciatica typically involves the removal of disc herniation or part of the disc, as well as addressing any stenosis causing nerve compromise. The primary goal of surgery is to address alarming symptoms including severe leg pain and muscle weakness. It is widely agreed that immediate surgery is necessary for cauda equina syndrome, while elective surgery is recommended for unilateral sciatica. A relatively old randomized trial indicates that surgical intervention may yield better outcomes after one year, but there are no significant differences in results after four and 10 years of follow-up [19, 20]. Moreover, previous literature demonstrated that patients who waited 12 weeks or more to undergo surgery reported worse pain 6 months post-surgery compared to those with a shorter waiting period [21]. A randomized controlled trial by Peul et al. was conducted to compare the outcomes of patients who received early lumbar disc surgery within 2 weeks versus those who underwent prolonged conservative treatment with later surgery if necessary [16]. The study found that although the overall outcomes were similar at 1 year, patients in the early surgery group experienced faster pain relief and global perceived recovery. Notably, the early treatment group had lower leg pain intensity during the first 6 months of follow-up. Additionally, a cost-utility analysis by van den Peul et al. from the same study revealed that while lumbar disc surgery was initially more expensive than non-surgical care, the benefits of faster recovery and reduced absenteeism from work ultimately offset the difference in costs [22].

A retrospective analysis of the SPORT data revealed that patients with a symptom duration of 6 months or longer experienced deleterious outcomes compared to those with a shorter duration, regardless of whether they received surgical or non-surgical treatment [21]. Specifically, patients who received early intervention (either surgery or conservative) within the first 6-12 weeks of symptom onset experienced greater pain relief, improved function, and higher satisfaction compared to those who underwent delayed treatment.

On the other hand, there are contraindications for surgery in chronic sciatica, including poor overall health, active infection, severe neurological deficits, coagulopathy, or bleeding disorders [21,22].

The main objective of non-surgical sciatica treatment is pain reduction, achieved either through the use of pain relievers or by reducing inflammation, which, in turn, alleviates pressure on the nerve root [15,22]. A systematic review study concluded that conservative treatments do not significantly alter the typical progression of sciatica in the majority of patients or alleviate symptoms [23]. Other treatment options for acute and chronic sciatica are intravenous and subcutaneous anti-TNF-α, which show very promising results in such patients [22]. Additionally, epidural steroid was recommended as a beneficial intervention for the disease due to its superior ability to reduce ODI [24]. It is important to note that non-surgical treatments are not always effective in treating the underlying cause of sciatica such as disc herniation or stenosis but rather focus on relieving symptoms such as pain, which is why they are defined as conservative treatment [25].

Sciatica can be influenced by various factors like nerve injury, inflammation, and disturbances in microglia [23]. After nerve injury, Schwann cells undergo important changes that help in fixing injured peripheral nerves [25]. Central sensitization is another potential factor as it amplifies neural signaling in the central nervous system, making people more sensitive to non-painful stimuli, and causing heightened pain responses. Symptoms linked to central sensitization, including widespread pain and sensitivity in nerves, can negatively impact a person's quality of life [26,27].

The severity of central sensitization, measured by the Central Sensitization Inventory (CSI), is significantly connected to symptoms before surgery and the quality of life in people having lumbar spine surgery [28]. Understanding these processes is vital for creating targeted therapies that adjust the inflammatory response. Since sciatic pain can last a long time and may involve more than just the initial cause, it's important to approach treatment in a careful and nuanced way. Considering this, it may be beneficial to assess risk factors before deciding between surgical and non-surgical options. For example, individuals with a history of mental stress may benefit more from conservative treatment, while those with occupational risk factors may benefit from a combination of conservative treatment and occupational advice before considering surgical options if symptoms persist.

The Optimal Timing for Surgical Intervention

A review conducted by Cochrane indicated that the long-term effects of surgical intervention for chronic sciatica are uncertain, and there is a lack of evidence regarding the best timing for surgery [29]. Several factors must be considered when determining the best timing for surgery in patients with chronic sciatica. These factors include the severity and longevity of symptoms, the underlying cause of the condition, the patient's overall health and functional status, and the potential risks and benefits of surgical intervention.

Additionally, recent studies have suggested that early surgical intervention may lead to better long-term outcomes for certain patients with chronic sciatica. The researchers concluded that early surgery may be more effective in relieving symptoms and preventing long-term disability in patients with severe and persistent sciatica [24].

On the other hand, some experts argue that a period of conservative management should be attempted before considering surgery for chronic sciatica. They suggest that many patients may experience spontaneous improvement in their symptoms with time and that surgical intervention should be reserved for those who fail to respond to noninvasive treatments. Additionally, delaying surgery allows for the identification and treatment of any reversible contributing factors, such as muscle imbalances or poor posture, which may alleviate symptoms without the need for invasive procedures.

However, some patients may prefer conservative over surgical treatment, especially if they think that conservative would be beneficial or if they have any concerns about the risk of surgery. For instance, although 40% of patients referred to spinal manipulative therapy for LDH-induced sciatica may fail to achieve satisfactory relief, the evident risk and cost profile associated with operative care argue for careful consideration by physicians and patients of spinal manipulative therapy before opting for surgical intervention [30].

Ultimately, the decision regarding the timing of surgery for chronic sciatica should be individualized based on each patient's unique circumstances. Healthcare providers should carefully assess the severity and duration of symptoms, the underlying cause of the condition, and the patient's overall health and functional status when determining the most appropriate course of treatment.

The Lack of Significant Effects of Age, Sex, and BMI on the Treatment Outcomes

Meta-regression analysis was conducted to explore the potential influence of age, sex, and BMI on the treatment effects. However, none of these variables were found to significantly affect the results. This suggests that the observed treatment effects are not influenced by these demographic or clinical characteristics. However, there may be other confounding factors that were not reported by our included studies [13, 14, 15, 16] that may influence the results, such as occupation.

Implications of the Findings for Clinical Practice and Decision-Making

International guidelines generally recommend surgical treatment for sciatica secondary to LDH if patients have not responded to comprehensive non-surgical treatment because surgery can provide more immediate and effective relief from symptoms [16]. Non-surgical treatments such as physical therapy, medication, and injections may not always provide sufficient relief for patients with severe or persistent symptoms. Surgery can directly address the underlying cause of the sciatica-nerve root compression-thereby reducing pain and improving function. Additionally, surgery may be recommended if there are signs of neurological deficits or if the patient's quality of life is significantly impacted by their symptoms. These recommendations are because many people with acute sciatica will have improvements in their condition over time [31]. But when it comes to chronic sciatica, the pain is already persisting for a long time, that's why surgical intervention should be considered if that's the case.

The Impact of the Study on the Existing Literature

To the best of our knowledge, this is the first systematic review and meta-analysis study that compares surgery versus non-surgical treatment while accounting for the clinical and prognostic differences in patients with chronic sciatica. Additional advantages include the standardized care that the non-surgical group's patients received, which included standardized chiropractic spine manipulation, analgesics, physiotherapy, and epidural steroid injections. In the Abd-Elaal et al. study [13], it was demonstrated that the crossover effect was reduced due to the extended wait time for a surgical appointment. Bailey et al. [14], in their secondary analysis, provided a relatively extended follow-up period to conduct an intention-to-treat analysis at the 2-year follow-up. Additionally, the nonoperative cohort received standardized treatment, and patients were excluded if the radiculopathy they presented with had been treated with the nonoperative modalities used in their trial, to avoid bias against nonoperative care. A further advantage of this trial is that patients randomized to nonoperative care received treatment from a separate study physician while on the surgeon’s waitlist. Surgery, if needed, happened after the usual 6-month wait, delaying crossover effects in our study compared to contemporary RCTs.

Limitations and potential sources of bias

One limitation of our study was the small number of included studies. Although we used a comprehensive search strategy, our search retrieved only three RCTs that included a small number of patients, thereby affecting the significance of our results. Another possible limitation of our study is the high heterogeneity observed in our results, despite our efforts to adjust for possible confounding factors and include only studies with similar designs (RCTs). Piriformis syndrome, on the other hand, can mimic chronic sciatica and cannot be determined based on lumbar MRI findings alone. It is necessary to mention the possibility that piriformis syndrome may be involved, and we must proceed with caution

The significant heterogeneity observed can be attributed to confounding factors such as work status and type of occupation. Unfortunately, we were unable to investigate their effects using meta-regression due to a lack of available data. Even when we were able to conduct meta-regression for age, sex, and pain duration variables, we found that they did not significantly affect our results.

Recommendations for future research

There is a need for multicenter global prospective RCTs, including patients with more than 4 months of symptoms, to compare the effectiveness of surgical and non-surgical approaches for persistent sciatica and to mitigate bias from genetic and physical factors among collaborators. These trials are crucial for addressing clinical uncertainties and optimizing patient care in real-time situations. Additionally, it is important to investigate the long-term outcomes of both surgical and conservative treatments for patients with persistent sciatica.

## Conclusions

Our findings suggest that surgical intervention may be more effective than non-surgical treatment for chronic sciatica-related back pain. Conservative treatment has been found to significantly reduce leg back pain and improve mental and physical health outcomes. However, its effects on leg pain reduction are less conclusive. It is essential to emphasize that conservative treatment should always be the initial approach unless surgery is warranted, such as in cases involving neurological deficits or cauda equina syndrome, as outlined in international guidelines. Therefore, for cases of chronic sciatica without neurological deficits, conservative treatment may be more appropriate for long-standing conditions. However, this result must be interpreted carefully, considering the significant heterogeneity observed, which in turn can be attributed to confounding factors that we could not adjust for.
